# The Use of the Emergency Department as a Place of Safety Following Section 136 Detention

**DOI:** 10.1192/bjo.2024.611

**Published:** 2024-08-01

**Authors:** Oluwadamilola Ogunsina, James Hickmott

**Affiliations:** Birmingham and Solihull Mental Health NHS Foundation Trust, Birmingham, United Kingdom

## Abstract

**Aims:**

Section 136 of the Mental Health Act 1983 allows for a person who appears to a constable to be suffering from a mental disorder and needing immediate care to be removed to a place of safety (POS) for their protection or the protection of others.

The legislation recommends that prior to making a decision to detain on Section 136, the constable must, if practicable, consult mental health services for information to guide decision making.

The 136 suite is the preferred POS except for patients requiring urgent medical treatment in which case the Emergency Department (ED) is preferred. If the 136 suite is unavailable, then alternatives like the ED may be used.

This audit examines the use of the Birmingham City Hospital Emergency Department as a POS following Section 136 detention, the adherence to the aforementioned legislation and the outcomes of the assessments.

**Methods:**

The audit was approved by the clinical governance team and a list of all Birmingham City Hospital patients detained under Section 136 for a three-month period (January–March 2022) was retrospectively obtained. Clinical records were examined, and the relevant data was extracted from the clinical notes.

Information including the reason for use of the ED as a POS, police contact with mental health services prior to detaining, time taken prior to assessment, reasons for mental health act assessment (MHAA) delays, and outcomes were collected and collated using Microsoft Excel.

**Results:**

The ED at City Hospital was used a place of safety for 80 patients in this period. In 52.5% of cases the ED was used as a place of safety due to lack of space at the POS. Contact with mental health services prior to detention was documented in only 29% of cases. The average time for a MHAA to take place in the period under review was 11.5 hours. Only 20% of these cases ended up detained under the mental health act.

**Conclusion:**

The results show poor adherence in the use of Section 136 to the recommendations of the legislation. Improvements are needed on time taken for assessments and use of ED as a place of safety due to unavailability of beds at the s136 suite. The police should be re-educated on the importance of contacting mental health services prior to detaining patients on Section 136. The audit result was presented at a clinical governance meeting and repeat audits are planned across all the emergency departments in Birmingham.

